# Injury of the Prefrontocaudate Tract in a Patient with a Bilateral Caudate Infarct

**DOI:** 10.4274/balkanmedj.2018.0201

**Published:** 2018-07-24

**Authors:** Sung Ho Jang, Hyeok Gyu Kwon

**Affiliations:** 1 Department of Physical Medicine and Rehabilitation, Yeungnam University College of Medicine, Gyeongsan, South Korea; 2 Department of Physical Therapy, College of Health Sciences, Catholic University of Pusan, Busan, South Korea

A 65-year-old female patient underwent graft interposition of the ascending aorta for acute aortic dissection. She was diagnoed with multiple cerebral infarction including bilateral caudate nuclei, bilateral frontotemporal lobes, and left parietal lobe after the operation (Figure 1a). At 4 weeks after onset, she was transferred to the rehabilitation department and showed severe abulia (decreased spontaneous activity and speech, disinterest, and flattened affect) with other associated symptoms (hypersomnia, severely impaired cognition, poor concentration, global aphasia, dysphonia, dysphasia, quadriparesis, depression, and ideational apraxia), which started after the operation. The patient provided written informed consent, and the study protocol was approved by the local research board.

Diffusion tensor imaging data were acquired at 4 weeks after onset using a 1.5-T magnetic resonance imaging (MRI) with 32 gradients. The Oxford Centre for Functional Magnetic Resonance Imaging of the Brain Diffusion Software with default options was used for eddy corrections and fiber tracking. This method makes 5.000 streamline samples from the seed region of interest with reflection of both dominant and nondominant orientations of diffusion in each voxel and presents the degree of connectivity in brain regions. For the caudate nucleus (CN) connectivity, the seed region of interest was placed on the CN, which is isolated by the adjacent structures (medial boundary: the lateral ventricle; lateral boundary: the anterior limb of the internal capsule) (1). A result threshold of 10 streamlines was applied. The neural connectivity of the CN to the medial prefrontal cortex [Brodmann area (BA): 10, 12] and the orbitofrontal cortex (BA: 11, 13) had disappeared in both hemispheres.

The CN is primarily involved in cognition as an important nucleus connecting the prefrontal cortex and the subcortical structures. Several studies have reported about the clinical features of the caudate infarct; these features are classified according to the following three different patterns of behavior abnormalities, which are the symptoms of the prefrontocaudate tract: 1) Abulia, 2) Disinhibition or impulsiveness, and 3) Affective symptoms such as depression and anxiety (2). However, the most common symptom of bilateral caudate infarct is abulia, which is consistent with the primary symptom of this patient (3,4).

In conclusion, injury of the prefrontocaudate tract was demonstrated in a patient who showed severe abulia following bilateral caudate infarction. Our results suggest that diffusion tensor tractography could provide useful information for detecting decreased connectivity of the prefrontocaudate tract, which could not be detected on conventional brain MRI in patients with stroke (Figure 1b).

## Figures and Tables

**Figure 1:**
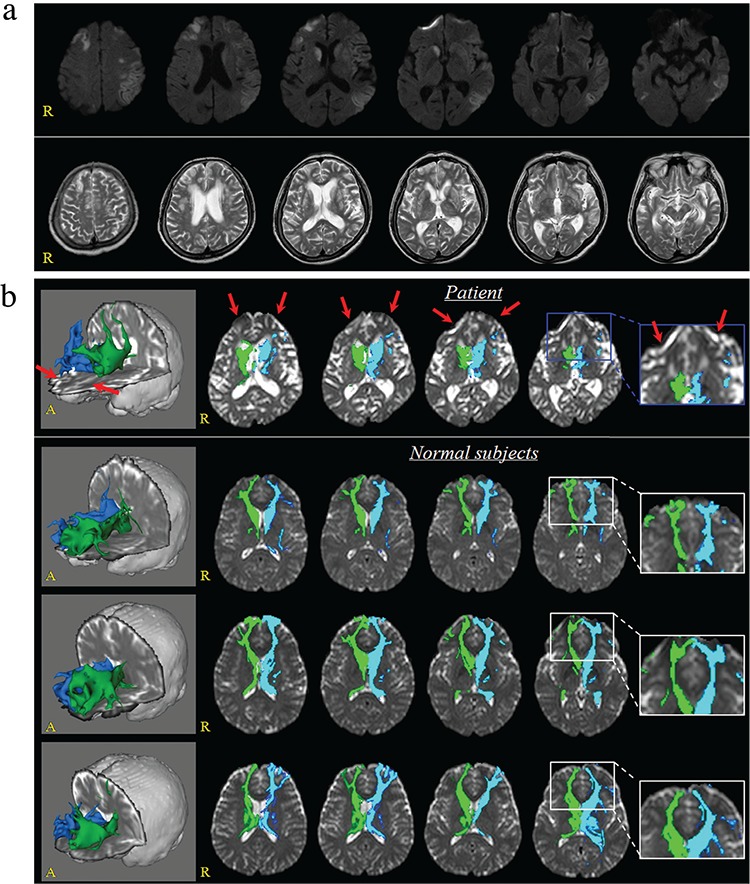
Diffusion (2 days after onset, upper low) and T2-weighted (4 weeks after onset, lower low) brain MR images showing multiple infarcts in the bilateral caudate nucleus, the bilateral frontotemporal lobes, and the left parietal lobe (a). The neural connectivity of the caudate nucleus to the prefrontal cortex (red arrows) is absent in both hemispheres compared to that in three normal subjects (three females, mean age: 62.3±3.5 years) (b).
